# Multimodal Remote Digital Phenotyping for Detecting and Tracking Early Parkinsonian Change in LRRK2 Carriers

**DOI:** 10.21203/rs.3.rs-9474934/v1

**Published:** 2026-05-08

**Authors:** T M Tariq Adnan, Abdelrahman Abdelkader, Md Saiful Islam, Meghan Pawlik, Emily Hartman, Ehsan Hoque, Ruth B Schneider

**Affiliations:** 1 Department of Computer Science, University of Rochester, Rochester, New York, United States.; 2 Department of Neurology, University of Rochester Medical Center, Rochester, New York, United States.; 3 Department of Computer Science and Engineering, Bangladesh University of Engineering & Technology, Dhaka, Bangladesh.; 4 Health Services, Ministry of Defense, Riyadh, Saudi Arabia.

## Abstract

Identifying prodromal Parkinson’s disease among *LRRK2* carriers is critical yet challenging. We present a remote, multimodal video framework analyzing 829 participants, including 158 *LRRK2* carriers (36 with manifest PD, 122 non-manifest), to address two key challenges: detecting high-risk carriers prior to clinical diagnosis and monitoring early disease-related change. Our AI model distinguished non-manifest carriers from controls with 92.9% accuracy (AUROC 0.92, AUPRC 0.82). Furthermore, our continuous *PD Weigh-In* score captured clinical decline in two carriers who subsequently developed PD and correlated strongly with expert ratings (Pearson *r* = **0.77**, Spearman ρ = **0.79**) across the held-out *LRRK2* test cohort.

Parkinson’s disease (PD) features a prolonged prodromal phase preceding the onset of classic motor symptoms [1, 2]. Identifying individuals during this pre-diagnostic window is expected to improve the likelihood of success in disease-modifying trials, which currently enroll patients after nearly 60% of dopaminergic neurons have already been lost [3]. However, traditional biomarkers (e.g., DAT imaging, a-synuclein assays) are burdensome and difficult to scale [4, 5], while standard clinical scales (e.g., MDS-UPDRS) [6] lack the sensitivity to detect subtle, pre-diagnostic motor changes. Clinical signs, such as REM sleep behavior disorder and hyposmia, may suggest increased risk for PD, however, these are not uniformly present at the earliest disease stages. Consequently, there is an urgent need for accessible, objective tools to identify high-risk individuals before overt motor symptoms manifest.

Genetically at-risk populations, particularly *LRRK2* G2019S carriers—the most common autosomal dominant genetic cause of PD [7, 8]—provide a unique opportunity to identify early biomarkers. Because penetrance is incomplete (e.g., only ~26% of Ashkenazi Jewish G2019S carriers develop PD by age 80 [9, 10]), longitudinal monitoring is essential to distinguish stable carriers from those actively transitioning to manifest disease (phenoconverters). Remote digital phenotyping offers a scalable solution. Consumer-grade cameras and microphones can capture granular kinematic and acoustic changes (hypomimia, tremors, dysarthria) that evade standard clinical observation [11, 12]. While previous remote phenotyping has successfully distinguished established PD from healthy controls [13–15], these binary models rely on gross motor deficits absent in the prodromal phase. A critical gap remains in detecting the “invisible” micro-signatures of prodromal disease in asymptomatic at-risk individuals and tracking their progression continuously.

In this study, we present a remote, multimodal video framework (Figure 1A) for the early characterization of *LRRK2* G2019S carriers through two distinct contributions. First, we adapt *UFNet,* our established multimodal fusion architecture [15], for the binary classification of non-manifest carriers versus controls. Second, we introduce *PD Weigh-In,* a continuous severity metric validated to capture clinical decline in phenoconverters and align strongly with expert-rated MDS-UPDRS III scores. Together, these tools provide a scalable, objective solution for early risk stratification and longitudinal monitoring in the prodromal phase.

We curated a multimodal dataset of 829 participants to capture prodromal motor signatures: 122 non-manifest and 36 manifest *LRRK2* G2019S carriers from the longitudinal VALOR-PD study [16, 17], plus 100 PD patients and 571 controls from complementary cohorts [18]. VALOR-PD participants (*N* = 158) completed annual, remotely supervised multimodal digital and clinical motor assessments, averaging 1.86±0.74 visits over 0.94± 0.81 years (maximum 2.34 years). Investigators rated motor severity using a modified MDS-UPDRS Part III (excluding rigidity and postural instability assessments; maximum score 108). Notably, two non-manifest carriers developed clinically manifest PD during follow-up. Pre-clinical carrier classification utilized the 122 non-manifest carriers (95% White, 58% female, 28% aged < 40 years) and 571 controls (85% White, 55% female, 11% aged < 40 years). While controls were not genetically tested, the low *LRRK2* G2019S prevalence (<1% in non-Ashkenazi populations) minimizes misclassification risk [7, 19]. For PD Weigh-In, we leveraged 304 participants with concurrent clinical scores: 158 from VALOR-PD and 146 from other cohorts (46 controls, 100 PD patients). This cohort was 44% PD, 40% *LRRK2* carriers, 51% female, 12% aged <40 years, and 95% White. Motor severity scores ranged from 0–67 (mean 12.2± 15.8), skewing toward prodromal/early stage (median 4.0, IQR 0–23). The University of Rochester and University of Rochester Medical Center IRBs approved all data collection.

Participants completed a standardized webcam and microphone protocol consisting of bilateral finger tapping, a smile expression, and a pangram utterance. These three tasks have been proven to be effective in capturing the core motor deficits of Parkinson’s disease: limb bradykinesia, facial hypomimia, and dysarthria [13–15, 20]. From the captured task videos, we extracted established, clinically interpretable computer vision features [13, 20]: 130 kinematic metrics (speed, amplitude, rhythm, interruptions) via MediaPipe [21] hand-tracking for tapping bradykinesia, and 42 Action Units (onset velocity and symmetry) via OpenFace [22] and MediaPipe for facial hypomimia. For speech audio, we utilized a dual-feature approach: 1024-dimensional self-supervised WavLM embeddings [23] for the binary classifier, and standard acoustic features (jitter, shimmer, fundamental frequency (*F*_*0*_) variability) serving as dysarthria and vocal tremor proxies [24, 25] for the interpretable PD Weigh-In metric.

To determine whether digital signatures can identify genetically at-risk individuals prior to clinical manifestation, we retrained UFNet—a multimodal fusion architecture originally developed for idiopathic PD classification [15]—to distinguish non-manifest *LRRK2* carriers from controls. UFNet dynamically integrates hand, facial, and speech features based on modality-specific certainty, optimizing the contribution of each task on a per-subject basis. Evaluating on the full cohort (693 participants; 122 carriers, 571 controls; total 806 samples) using a 60–20-20 subject-stratified split, the model demonstrated robust discrimination on the held-out test set (*N* = 155 samples) with an AUROC of 0.92 ± 0.06. Crucially, addressing the low carrier prevalence (20% at sample-level), the model achieved an AUPRC of 0.82 ± 0.13, vastly exceeding the 0.20 random baseline. To mitigate demographic confounding while preserving sufficient neural network training volume, we evaluated a hierarchically matched 1:1.5 cohort (122 carriers, 183 controls; matched on age, sex, race). By deliberately maintaining a near-balanced held-out test set (48% carriers), we ensured unbiased evaluation, yielding high performance (AUROC 0.92 ± 0.06, AUPRC 0.91 ± 0.12). While excluding demographically distinct controls slightly lowered specificity (81.6 ± 12.4%), sensitivity improved (91.4 ± 9.1%). Consistent positive predictive values across the full (82.8±14.3%) and matched (82.1±12.3%) cohorts confirm the digital phenotype’s robustness. We further conducted a subgroup analysis in the full cohort performance to ensure equitable performance. The model exhibited no statistically significant misclassification bias across sex (two-proportion z-test, FDR-corrected p = 1.00; male: 7.4%, 5/68; female: 6.9%, 6/87) or racial categories (Fisher’s exact test, FDR-corrected p = 1.00; White: 7.4%, 10/135; Non-White: 5.0%, 1/20). Misclassification varied slightly by age, with the lowest error rate in the oldest cohort (60–79 years: 2.5%, 2/80) compared to younger participants (20–39 years: 14.3%, 3/21; 40–59 years: 9.0%, 4/44); however, this variance was not statistically significant (χ^2^ test, FDR-corrected *p* = 0.26). Finally, Monte Carlo dropout-based uncertainty estimation revealed informative uncertainty stratification. Across the full and demographically matched cohorts combined, test samples stratified by predictive standard deviation showed the highest accuracy in the low-uncertainty group (95.4 ± 3.4%, *n* = 146), while accuracy remained strong even in the high-uncertainty group (90.1 ± 12.5%, *n* = 13). Full metrics and subgroup analyses are detailed in Figure 1.

To develop PD Weigh-In, we subject-stratified 304 participants (158 VALOR-PD, 146 complementary) into an 80% development and 20% held-out test set. Using 5-fold cross-validation on the training partition, we evaluated diverse regression architectures: linear models (Ridge, Lasso, Elas-ticNet, Bayesian Ridge), ensembles (Random Forest, Extra Trees, XGBoost, CatBoost, LightGBM), and a Multi-Layer Perceptron. Extra Trees emerged as the optimal architecture. Evaluated on the independent test set (70 participants, 149 samples), the model achieved an *R*^2^ of 0.76, an RMSE of 8.34, and an MAE of 5.69 (a ~5.3% relative error given the 0–108 modified MDS-UPDRS Part III range). Predicted scores demonstrated strong agreement with clinical ground truth (Pearson *r* = 0.89, Spearman *ρ* = 0.90). To assess performance on the target *LRRK2* population, we performed a split-sample validation, reserving 40 VALOR-PD participants (81 samples, including two phenoconverters) as an independent test set. The model maintained robust performance on this heldout cohort (Pearson *r* = 0.77, Spearman *ρ*= 0.79, *R*^2^ = 0.55). Performance across both cohorts is detailed in Figure 2A,B.

Longitudinal analysis of the 40 held-out VALOR-PD participants tracked individual disease trajectories over 2+ years on the MDS-UPDRS Part III scores (modified scale: 0–108), capturing both stable and progressive courses. Notably, the model captured the severity progression of two participants who phenoconverted from non-manifest carriers to clinical PD during the study (Figure 2C). For both phenoconverters, predicted PD Weigh-In scores increased from ≈ 1 at baseline to 16 at diagnosis, mirroring their respective clinical score escalations (Phenoconverter A: 2 to 11; Phenoconverter B: 0 to 5). Across additional representative participants (Figure 2D), predicted scores consistently tracked the overall direction and relative changes of clinical MDS-UPDRS Part III scores over time for both stable and progressively worsening cases.

We analyzed feature importance using SHAP [26] to identify the most salient predictive biomarkers (Figure 3). For *LRRK2* carrier classification via UFNet, speech features (primarily high-dimensional, non-semantic WavLM embeddings) provided the highest global importance, followed by finger tapping and facial expressions. Top motor predictors included reduced rhythmic consistency, increased temporal irregularity, and declining amplitude in tapping, alongside reduced dynamic range in mouth/eye opening and zygomaticus major (Action Unit 12) activation. For the PD Weigh-In regression framework, SHAP identified distinct modality-specific biomarkers driving higher predicted severity. The most influential facial feature was reduced variability in the zygomaticus major muscle (AU12), indicating diminished expressivity. Top acoustic measures included Mel-frequency cepstral coefficients and Teager energy features, reflecting subtle degradation in vocal control and articulation. Finally, for finger tapping, higher inter-tap interval entropy in both hands strongly predicted higher MDS-UPDRS scores, directly aligning with the clinical manifestation of bradykinesia.

Parkinson’s disease (PD) currently lacks disease-modifying treatments. Intervening earlier, before a clinical diagnosis of PD, may increase the chances of preserving neuronal function and altering disease trajectories. To enable earlier intervention, we need new, scalable strategies that support the identification of at-risk individuals. Traditional biomarkers (e.g., DAT imaging, a-synuclein assays) are burdensome and expensive, limiting their longitudinal utility. This study provides evidence that remote, multimodal digital phenotyping via consumer-grade devices may help address these barriers by detecting subtle motor signatures in a genetically at-risk group of non-manifest *LRRK2* G2019S carriers. By combining binary risk stratification (AUROC 0.92) with continuous severity monitoring (PD Weigh-In, Pearson *r* = 0.89), we propose a scalable framework that could support early intervention trials.

The carrier-screening model captures measurable preclinical differences. Sustained high PPV and sensitivity across full and demographically matched cohorts, supported by the absence of statistically significant misclassification bias across sex, race, and age subgroups, suggests these discriminative signals are not primarily driven by demographic confounders. However, subgroup analyses should be interpreted cautiously given the limited subgroup sample sizes, particularly the low representation of non-White participants. Furthermore, uncertainty stratification showed that low-uncertainty predictions were the most accurate, supporting the practical usefulness of the model’s uncertainty estimates. While exact biological mechanisms require further study, these signals align with the subtle functional alterations anticipated with genetic risk. Complementing this risk stratification, PD Weigh-In provides continuous motor severity estimates that align with clinical assessments. The model’s MAE of 5.69 (~5.3% of the modified 0–108 scale) is of the same order of magnitude as published estimates of the Minimal Clinically Important Difference (3.25–4.6 points) for the MDS-UPDRS Part III [27], suggesting potential utility for longitudinal tracking.

Importantly, it maintained meaningful correlations (*r* = 0.77) within the restricted, prodromal or early-stage VALOR-PD cohort. Longitudinally, predicted trajectories mirrored clinical trends across stable and progressive cases, including two observed phenoconverters. Though limited conversion events preclude definitive lead-time conclusions, these concordant trajectories support the feasibility of digital biomarkers for tracking disease progression and potentially detecting early transition signals.

Clinical translation benefits from transparency. SHAP analysis revealed distinct modality contributions: speech features drove carrier classification, while facial AU12 variance (hypomimia) was the strongest severity predictor in PD Weigh-In. By quantifying interpretable signs of bradykinesia, hypomimia, and dysarthria, our standardized protocol (finger tapping, smile, pangram) offers mechanistic insights rather than entirely opaque predictions.

A key advantage is compatibility with widely available hardware, enabling fully remote assessments without specialized equipment. By pairing a standardized protocol (finger tapping, smile, pangram) with the ecological feasibility of repeated home monitoring, this scalable framework may facilitate the assessment of geographically dispersed populations and could potentially extend to low-burden, smartphone-based testing.

Several limitations warrant consideration. Controls were not genetically tested, although the low population prevalence of *LRRK2* G2019S reduces the likelihood of substantial misclassification. The cohort lacked racial diversity (95% White), limiting immediate generalizability. Additionally, the modified MDS-UPDRS excludes rigidity and postural instability assessments that cannot be performed remotely, meaning the digital ground truth metric does not encompass the full motor examination. Moreover, although the predicted score is reported on a 0–108 scale, it is inferred from only a limited set of remote tasks rather than direct assessment of all 27 constituent motor items, and should therefore be interpreted as a task-informed proxy of overall motor severity rather than a full item-by-item reconstruction of the clinical examination. Finally, observing only two phenoconverters restricts inferences regarding predictive timing.

Despite these limitations, our findings establish proof-of-principle for digital phenotyping in populations genetically at-risk for PD. As clinical trials increasingly target the pre-diagnostic phase, scalable digital biomarkers may become valuable tools for participant selection, sensitive outcome measurement, and dynamically adjusting monitoring intensity or trial interventions based on longitudinal severity changes.

## Methods

### Data Sources

Data for this study were aggregated from several prior remote and community-based PARK studies that used a common multimodal assessment protocol, including finger tapping, smile, and speech tasks. The cohort combined participants from both genetically enriched and general-population studies to support carrier screening and severity modeling.

**VALOR-PD** contributed 158 *LRRK2* G2019S carriers, including 122 non-manifest carriers and 36 manifest participants with PD. This longitudinal substudy enrolled *LRRK2 G2019S carriers* from the parent VALOR-PD cohort and collected annual remote assessments under study supervision as needed.

**ParkTest** contributed 412 control participants through a publicly accessible web-based platform for remote PD-related task collection. This study enrolled individuals with and without self-reported PD and enabled large-scale home-based data acquisition.

**SuperPD** contributed 98 participants from a clinically supervised longitudinal study in the Rochester, New York area, including 35 controls and 63 participants with clinically confirmed PD.

**Cluster-PD** contributed 57 control participants from a supervised clinical study examining environmental exposure and PD risk.

**ParkTest@InMotion** and two subsequent validation studies contributed 67 additional control participants collected in supervised community and unsupervised home settings, primarily through recruitment of caregivers and participants without diagnosed movement disorders.

**ROUTE-PD** contributed 27 participants with clinically confirmed PD from a longitudinal community-based study designed to track symptom progression over time.

All contributing studies received approval from the relevant institutional review board(s), and all participants provided informed consent.

### Clinical Reference Standard

Motor severity labels were derived from clinician-administered MDS-UPDRS Part III assessments aligned with the multimodal recordings. For VALOR-PD, investigators completed a remotely administered modified Part III examination in which rigidity and postural instability items were omitted because they cannot be assessed reliably in the remote setting. For SuperPD and ROUTE-PD, participants underwent standard in-person MDS-UPDRS Part III evaluations on the full 132-point scale. To harmonize severity labels across cohorts, we removed the rigidity- and postural-instability-related items from these assessments and converted them to the same modified Part III scale used in VALOR-PD. The final reference score therefore corresponded to a remote-compatible modified MDS-UPDRS Part III total with a maximum possible score of 108 for all participants included in severity modeling. This harmonized modified score served as the clinical reference standard for development and evaluation of the *PD Weigh-In* continuous severity model.

### Task Selection for Remote Assessment Protocol and Feature Extraction

To capture complementary manifestations of Parkinsonian impairment, we analyzed three standardized tasks spanning motor, facial, and speech modalities: finger tapping, smile mimicry, and pangram utterance. In the finger-tapping task, participants tapped their thumb and index finger together 10 times as quickly and as largely as possible. In the smile task, participants smiled naturally and returned to a neutral expression, repeating the sequence three times. In the speech task, participants read aloud the pangram, “The quick brown fox jumps over the lazy dog.” These tasks were selected for their clinical relevance to bradykinesia, hypomimia, and speech impairment, as well as their practicality for remote assessment. In contrast to gait-based evaluations or other more environment-sensitive protocols, they can be completed safely while seated using standard home devices and are well suited for scalable, unsupervised data collection [13–15, 25]. Participants contributed data by recording themselves with a computer webcam while performing these three tasks on our web-based data collection platform, PARK (https://parktest.net) [28].

Because the dataset size and recording conditions were not optimal for fully end-to-end modeling from raw signals, we extracted structured task-specific features informed by prior work. For finger tapping, MediaPipe [21] was used to derive 130 kinematic features across both hands, capturing movement speed, amplitude, rhythm, and interruptions. For smile videos, OpenFace [22] and MediaPipe were used to extract 42 facial features reflecting movement intensity, spontaneity, blinking, and lip dynamics. For speech, we used different representations for the two downstream tasks. For carrier classification, we extracted 1024-dimensional WavLM embeddings [23], which capture rich high-level acoustic patterns related to articulation, prosody, and vocal quality. For continuous severity modeling, we instead used a set of interpretable handcrafted acoustic features, including measures such as pitch variability, jitter, and shimmer, to better preserve clinical interpretability of the predicted severity signals. These multimodal representations served as inputs for downstream carrier classification and continuous severity modeling.

### Carrier Classification Model Training and Evaluation

To identify subtle prodromal digital signatures associated with *LRRK2* carrier status, we adapted our previously developed Uncertainty-calibrated Fusion Network (UFNet) [15] for binary classification of non-manifest (not yet developed clinically diagnosed PD) carriers versus controls. The model followed the same overall multimodal fusion framework as in prior work, but was retrained here for the carrier-screening task using the feature representations described above. UFNet uses a two-stage architecture. In the first stage, task-specific models were trained separately for finger tapping, smile, and speech using the corresponding modality features. In the second stage, the learned task-level representations and predictions were combined through an uncertainty-aware fusion module that integrates information across modalities while downweighting less reliable task-specific signals. This design was particularly useful in our setting, where the strength and quality of behavioral markers may vary across individuals and modalities.

Model development was performed using subject-stratified splits to prevent leakage across repeated observations from the same individual. In the primary analysis, the full carrier-screening cohort (122 non-manifest carriers and 571 controls) was divided into 60% training, 20% validation, and 20% held-out test partitions at the participant level. Hyperparameter optimization for both the modality-specific subnetworks and the fusion model was conducted on the validation set using Weights & Biases (W&B; wandb) (https://wandb.ai) to manage experiment tracking and model selection. After selecting the best-performing configuration, the final model was locked and evaluated once on the held-out test set.

Because the full cohort was demographically imbalanced and contained substantially more controls than carriers, we performed an additional evaluation on a hierarchically matched cohort with a target 1:1.5 carrier-to-control ratio (122 carriers, 183 controls). Controls were matched to carriers without reuse through a sequential procedure that prioritized exact matching on sex, race, and age group, and then progressively relaxed the criteria to sex-and-race matching and sex-only matching when exact matches were unavailable. This secondary analysis was designed to reduce demographic confounding while preserving sufficient sample size for multimodal model evaluation.

Model performance was assessed using metrics relevant to imbalanced clinical screening, including area under the receiver operating characteristic curve (AUROC), area under the precision-recall curve (AUPRC), accuracy, sensitivity, specificity, positive predictive value, negative predictive value, and F1 score. Confidence intervals for held-out test performance were estimated by bootstrapping. In addition, because UFNet uses Monte Carlo dropout at inference, we examined predictive uncertainty by stratifying test samples according to predictive standard deviation and comparing performance across uncertainty bands. This analysis allowed us to determine whether the model’s most confident predictions were also its most reliable, and whether any samples fell into a high-uncertainty range.

### Development and Evaluation of *PD Weigh-In*

We developed *PD Weigh-In* as a continuous multimodal severity model to estimate the harmonized modified MDS-UPDRS Part III score from the remote task features. The model learns this score from multimodal features derived from bilateral finger tapping, smile, and pangram speech recordings, using these remote assessments as compressed behavioral surrogates of the broader modified MDS-UPDRS Part III construct. Model development used the subset of 304 participants with concurrent clinical reference scores, including 158 participants from VALOR-PD and 146 participants from complementary cohorts. To prevent leakage across repeated visits, data were partitioned at the participant level into an 80% development set and a 20% held-out test set.

Within the development partition, we evaluated a diverse set of regression models using 5-fold cross-validation, including linear approaches (Ridge, Lasso, ElasticNet, Bayesian Ridge), ensemble methods (Random Forest, Extra Trees, XGBoost, CatBoost, LightGBM), and a multilayer perceptron. Hyperparameters were tuned using grid search, with model selection based on cross-validated root mean squared error. The final model was then refit on the full development set and evaluated once on the held-out test cohort. In addition to the multimodal task features, the regression framework incorporated participant-level covariates available at assessment, including age, sex, and race.

Extra Trees achieved the best overall performance during model selection and was therefore used as the final *PD Weigh-In* model. Predictive performance on held-out data was assessed using coefficient of determination (R^2^), root mean squared error (RMSE), mean absolute error (MAE), Pearson correlation, and Spearman correlation between predicted and clinician-rated scores.

To assess transportability to the target genetically at-risk population, we performed an additional split-sample validation restricted to VALOR-PD, reserving 40 VALOR-PD participants (81 samples, including the two phenoconverters) as an independent test cohort for severity evaluation. This analysis was designed to determine whether a model trained on a broader severity distribution retained predictive signal in a predominantly prodromal and early-manifest *LRRK2* population. Longitudinal analyses were then performed within this held-out VALOR-PD cohort by comparing predicted and observed modified MDS-UPDRS Part III scores across visits, including the two individuals who phenoconverted from non-manifest carrier status to clinically manifest Parkinson’s disease during follow-up.

### Statistical Analysis

Performance metrics for the carrier classification task were quantified using 10,000 bootstrap resampling with replacement. For each metric, we estimated the mean and standard error across bootstrap samples and reported 95% confidence intervals using a normal approximation (*μ* ± 1.96 × SE). In the manuscript, metrics are reported in mean ± confidence interval format.

Subgroup robustness was evaluated in the held-out carrier-screening cohort by comparing *mis-classification rates* across demographic strata. For each subgroup, we summarized the error rate together with its bootstrap mean, standard error, and 95% confidence interval. Sex-based differences in error rates were assessed using a two-proportion z-test when standard large-sample conditions were satisfied. Race-based differences were assessed using Fisher’s exact test because of small cell counts in the non-White subgroup. Age-group differences were assessed using a chi-square test of independence across the prespecified age strata 20–39, 40–59, and 60–79 years. P-values from the sex, race, and age-group analyses were further adjusted using the Benjamini–Hochberg false discovery rate procedure.

### Model Explainability Analysis

To improve interpretability of the developed models, we performed post hoc explainability analysis using SHAP (SHapley Additive explanations) [26]. Separate SHAP analyses were conducted for the carrier-classification model and the *PD Weigh-In* severity model. For the carrier-classification model, SHAP was used to assess the relative importance of speech, finger-tapping, and smile-derived inputs, as well as to identify the most influential features within each modality. Feature-level importance within the finger-tapping and facial modalities was summarized by ranking variables according to their average absolute SHAP value across evaluated samples. Because the speech branch for this task was based on high-dimensional WavLM embeddings, individual speech dimensions were treated as latent coordinates and interpreted primarily at the aggregate modality level rather than as semantically meaningful standalone features.

For *PD Weigh-In*, SHAP was used to identify the features most strongly associated with higher predicted motor severity. Explanations were summarized separately for speech, finger tapping, and facial features by ranking variables according to mean absolute SHAP value.

## Figures and Tables

**Fig. 1:**
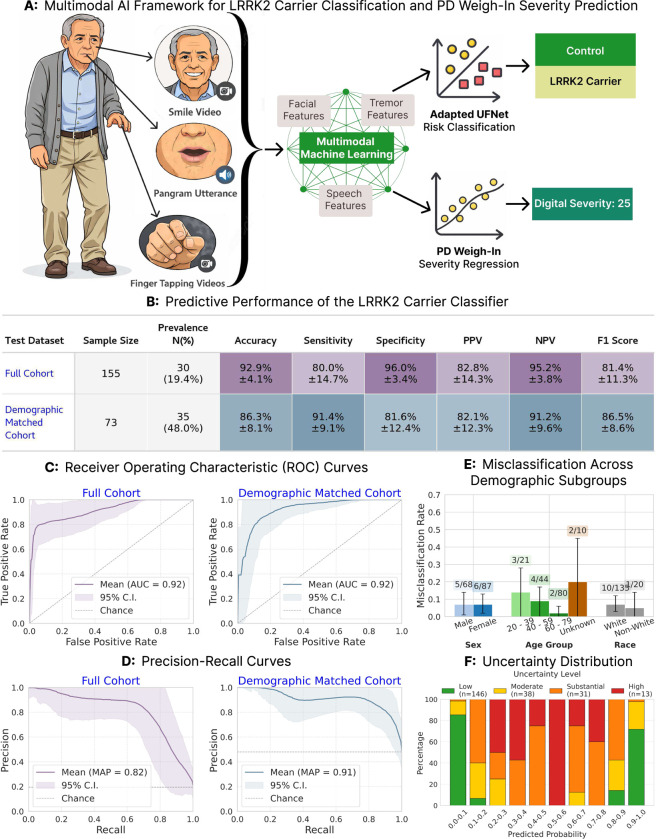
Framework overview, predictive performance, subgroup robustness, and uncertainty characterization of the LRRK2 carrier screening model. ***A:*** Overview of the proposed multimodal screening framework. Smile videos, pangram utterances, and finger tapping videos are used to extract facial, speech, and bradykinesia-related kinematic features respectively, which are combined through a multimodal machine learning model to predict both *LRRK2* carrier status (Control vs. *LRRK2* Carrier) and a continuous digital severity score. ***B:*** Performance metrics on held-out test sets for the full cohort (*N* = 155 samples, 19.4% carrier prevalence) and demographically matched cohort (*N* = 73 samples, 48.0% carrier prevalence). Values represent mean ± 95% CI across 10,000 bootstrapped runs. ***C:*** Receiver Operating Characteristic (ROC) curves showing strong discrimination in both the full cohort (AUROC = 0.92) and matched cohort (AUROC = 0.92). Shaded regions indicate 95% confidence intervals. ***D:*** Precision-Recall curves for both cohorts, achieving mean average precision (MAP) of 0.82 and 0.91, substantially exceeding the random baseline (dashed line at prevalence rate). ***E:*** Misclassification rate stratified by demographic subgroups (sex, age, and race) in the full cohort, demonstrating balanced performance across demographics. Error bars indicate 95% confidence intervals; numbers above bars show misclassified/total samples per group. ***F:*** Uncertainty stratification analysis on full and matched cohort combined showing model uncertainty across predicted probability bins, with uncertainty levels categorized as low (green), fair (yellow), substantial (orange), and high (red). The model demonstrates informative uncertainty stratification across the probability spectrumŵith the most extreme probability bins showing a higher proportion of low-uncertainty predictions.

**Fig. 2:**
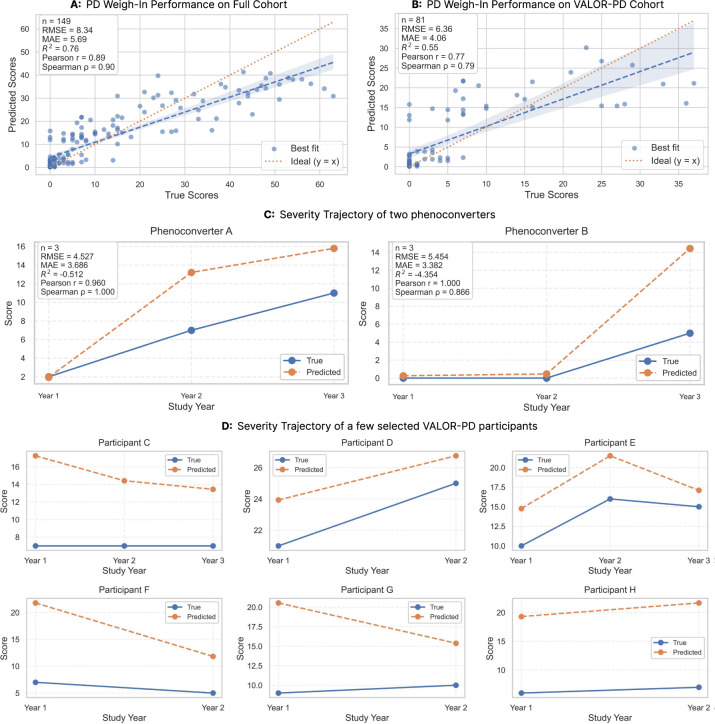
Performance and Longitudinal Validation of PD Weigh-In Framework. ***A:*** Predicted versus true MDS-UPDRS Part III scores on the held-out test set from the full cohort (*n* = 149 samples). The model achieved strong performance with *R*^*2*^ = 0.76, RMSE=8.34, MAE=5.69, Pearson *r* = 0.89, and Spearman *ρ* = 0.90. Blue dashed line shows best fit; orange dotted line indicates ideal prediction (y = x). Shaded region represents 95% confidence interval. ***B:*** Performance on the held-out VALOR-PD cohort (*n* = 81 samples from 40 participants). Despite the restricted severity range in this predominantly pre-manifest and early-manifest population, the model maintained robust correlations (Pearson *r* = 0.77, Spearman *ρ* = 0.79) with *R*^2^ = 0.55, RMSE=6.36, and MAE=4.06. ***C:*** Longitudinal tracking of the two phenoconverters who transitioned from non-manifest carrier status to clinical PD diagnosis during the study. ***Left:*** Phenoconverter A showed progressive increase in both predicted (orange) and true (blue) scores over two years, with predicted scores rising from ≈ 2 to 16 and clinical scores from 2 to 11. ***Right:*** Phenoconverter B exhibited gradual progression with predicted scores increasing from ≈ 0 to 15 and clinical scores from 0 to 5, demonstrating the model’s ability to capture subtle disease progression in the prodromal-to-manifest transition. ***D:*** Additional representative longitudinal trajectories from six VALOR-PD participants suggest that PD Weigh-In captures subject-level differences in motor burden while remaining sensitive to within-subject change. Across participants with varying baseline severity and progression patterns, predicted scores generally tracked the direction of clinical change, although predictions were often shifted upward relative to clinician-rated scores, reflecting a tendency toward overestimation in this lower-severity cohort. Visit years were anonymized as relative study years to protect participant privacy.

**Fig. 3:**
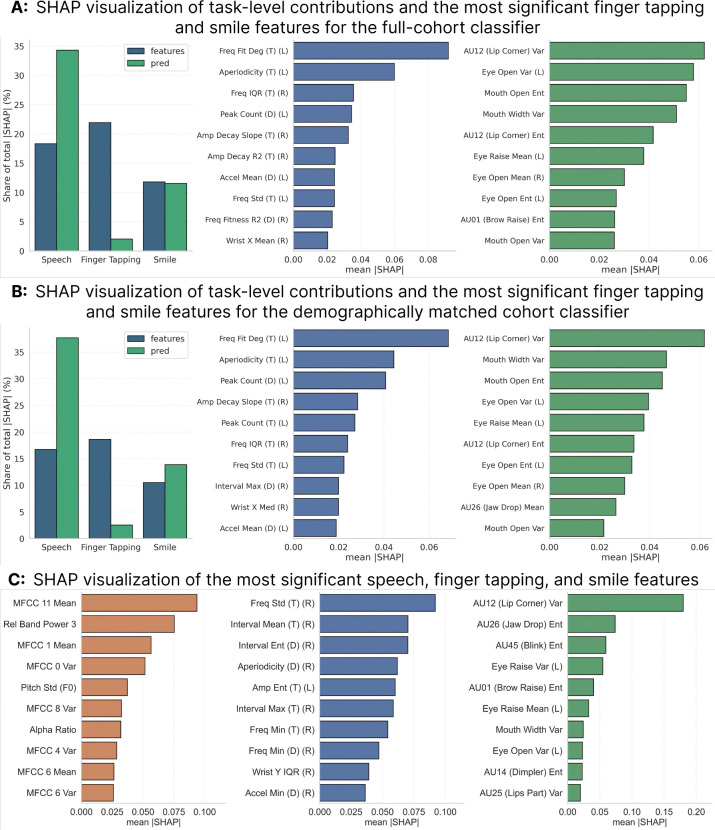
Feature importance analysis for carrier classification and PD Weigh-In models. ***A:*** SHAP analysis for UFNet carrier classification in the full cohort (*N* = 693). ***Left:*** Modality-level contribution shows speech features account for the largest share of total importance (~ 35%), followed by finger tapping (~ 22%) and smile (~ 12%). ***Middle:*** Top finger tapping features emphasize frequency fit characteristics, aperiodicity, peak count, and amplitude decay metrics, reflecting disruptions in tapping rhythm and movement consistency. ***Right:*** Top facial features are dominated by variability and entropy of zygomaticus major (AU12), eye opening, and mouth opening measures. ***B:*** SHAP analysis for the demographically matched cohort (*N* = 305) demonstrates consistent modality-level contributions and feature rankings, with speech remaining the dominant contributor (~ 38%), confirming stability across cohort compositions. ***C:*** SHAP analysis for the PD Weigh-In regression model. ***Left:*** Speech features are led by Mel-frequency cepstral coefficients (e.g., MFCC-1, MFCC-11), relative band power, and pitch variability, capturing changes in vocal energy and articulation. ***Middle:*** Finger tapping features highlight movement regularity metrics, including frequency variability, interval statistics, and aperiodicity. ***Right:*** Facial features are strongly driven by reduced AU12 variance, with additional contributions from jaw drop (AU26), blink (AU45), and mouth variability measures, indicating diminished facial expressivity associated with higher motor severity.

**Table 1:** List of IRB-approved study protocols.

Studies	Approving Institution	IRB Number

VALOR-PD	University of Rochester Medical Center	RSRB#00003703
SuperPD	University of Rochester Medical Center	RSRB#00001787
Cluster-PD	University of Rochester Medical Center	RSRB#00004849
Parktest, Parktest@InMotion	University of Rochester	RSRB#00001369
ROUTE-PD	University of Rochester	RSRB#00008275

## Data Availability

The data used in this study consist of participant video recordings collected through our web-based platform, available at https://parktest.net [28] under multiple IRB-approved studies. Because these recordings contain identifiable participant information, we are not able to release the raw video data publicly in accordance with IRB restrictions and the Health Insurance Portability and Accountability Act (HIPAA). Access may be considered for collaborative research purposes on a case-by-case basis, subject to approval under the applicable IRB protocols and completion of a data use agreement. Requests should be directed to the principal investigator, Ehsan Hoque (mehoque@cs.rochester.edu). In contrast, the derived tabular feature sets used for model development and analysis in this work are publicly available at https://github.com/ROC-HCI/parkinsons-risk-stratification.

## Code availability

The code used for model development, evaluation, and figure generation in this study is publicly available at https://github.com/ROC-HCI/parkinsons-risk-stratification. Related UFNet dataset resources and preprocessing code are also available at https://github.com/ROC-HCI/PARK.
